# Re-evaluation of the *nor* mutation and the role of the NAC-NOR transcription factor in tomato fruit ripening

**DOI:** 10.1093/jxb/eraa131

**Published:** 2020-04-27

**Authors:** Ying Gao, Wei Wei, Zhongqi Fan, Xiaodan Zhao, Yiping Zhang, Yuan Jing, Benzhong Zhu, Hongliang Zhu, Wei Shan, Jianye Chen, Donald Grierson, Yunbo Luo, Tomislav Jemrić, Cai-Zhong Jiang, Da-Qi Fu

**Affiliations:** 1 Laboratory of Fruit Biology, College of Food Science & Nutritional Engineering, China Agricultural University, Beijing, China; 2 College of Agriculture & Biotechnology, Zhejiang University, Hangzhou, China; 3 State Key Laboratory for Conservation and Utilization of Subtropical Agro-bioresources/Guangdong Provincial Key Laboratory of Postharvest Science of Fruits and Vegetables, College of Horticulture, South China Agricultural University, Guangzhou, China; 4 School of Food and Chemical Engineering, Beijing Technology and Business University, Beijing, China; 5 Plant Sciences Division, School of Biosciences, University of Nottingham, Sutton Bonington Campus, Loughborough, UK; 6 Department of Pomology, Faculty of Agriculture, University of Zagreb, Zagreb, Croatia; 7 Department of Plant Sciences, University of California, Davis, CA, USA; 8 Crops Pathology and Genetics Research Unit, United States Department of Agriculture, Agricultural Research Service, Davis, CA, USA; 9 Fondazione Edmund Mach, Italy

**Keywords:** CRISPR/Cas9, gene function, *NAC-NOR*, *nor* mutant, tomato fruit ripening, transcription factor

## Abstract

The tomato *non-ripening* (*nor*) mutant generates a truncated 186-amino-acid protein (NOR186) and has been demonstrated previously to be a gain-of-function mutant. Here, we provide more evidence to support this view and answer the open question of whether the *NAC-NOR* gene is important in fruit ripening. Overexpression of *NAC*-*NOR* in the *nor* mutant did not restore the full ripening phenotype. Further analysis showed that the truncated NOR186 protein is located in the nucleus and binds to but does not activate the promoters of 1-aminocyclopropane-1-carboxylic acid synthase2 (*SlACS2*), geranylgeranyl diphosphate synthase2 (*SlGgpps2*), and pectate lyase (*SlPL*), which are involved in ethylene biosynthesis, carotenoid accumulation, and fruit softening, respectively. The activation of the promoters by the wild-type NOR protein can be inhibited by the mutant NOR186 protein. On the other hand, ethylene synthesis, carotenoid accumulation, and fruit softening were significantly inhibited in CR-NOR (CRISPR/Cas9-edited *NAC-NOR*) fruit compared with the wild-type, but much less severely affected than in the *nor* mutant, while they were accelerated in OE-NOR (overexpressed *NAC-NOR*) fruit. These data further indicated that *nor* is a gain-of-function mutation and *NAC-NOR* plays a significant role in ripening of wild-type fruit.

## Teaser

The 186 aa truncated protein produced by the tomato spontaneous non-ripening (*nor*) mutant enters the nucleus and combines with the promoters of its target genes, resulting in a gain of function.

## Introduction

Plant fruits protect developing seeds and aid in their dissemination (Forlani *et al.*, 2019). They are also an important food source for humans and animals and are rich in nutrients such as carbohydrates, fats, proteins, vitamins, and trace elements (Giovannoni, 2004; Klee and Giovannoni, 2011). Fleshy fruit ripening and the generation of quality attributes occur towards the end of seed development and render fruit attractive to animal and human consumers, further aiding seed dispersal (Klee and Giovannoni, 2011). Understanding fruit ripening provides an important theoretical and practical basis for manipulating the ripening process, improving fruit quality, and prolonging fruit shelf life (Gómez *et al.*, 2014; Pesaresi *et al.*, 2014).

Tomato is a model plant for studying the ripening of climacteric fruit because of its simple diploid genetics, small genome size (approximately 950 Mb), short life cycle, ease of transient and stable transformation, distinct ripening phenotypes, and abundant bioinformatics resources (Alexander and Grierson, 2002; Giovannoni, 2004; Klee and Giovannoni, 2011). Molecular genetics studies have shown that tomato fruit ripening is governed by a transcription regulation network that is coordinated by a series of ripening-related transcription factors (TFs) (Karlova *et al.*, 2014; Liu *et al.*, 2015; Giovannoni *et al.*, 2017; Li *et al.*, 2019) and ethylene (Alexander and Grierson, 2002; Li *et al.*, 2019). Exploring the roles of these ripening-related TFs is an effective tool for understanding the mechanisms involved in fruit ripening. Tomato has abundant natural mutants (Giovannoni, 2007), some of which have obvious ripening-inhibited phenotypes, such as *ripening-inhibitor* (*rin*) (Vrebalov *et al.*, 2002), *non-ripening* (*nor*) (Mizrahi *et al.*, 1976; White, 2002; Kumar *et al.*, 2018), *Colorless non-ripening* (*Cnr*) (Eriksson *et al.*, 2004; Manning *et al.*, 2006), *Never-ripe* (*Nr*) (Lanahan *et al.*, 1994; Wilkinson *et al.*, 1995; Yen *et al.*, 1995), and *Green-ripe* (*Gr*) (Barry *et al.*, 2005; Barry and Giovannoni, 2006). *Nr* and *Gr* are related to ethylene signal transduction, while *rin*, *Cnr*, and *nor* TFs are involved in the transcription regulation network controlling the expression of tomato fruit ripening-related genes that determines quality attributes. However, several detailed studies of *rin*, *Cnr*, and *nor* mutants involving CRISPR/Cas9 gene editing have caused the roles of these mutants to be re-evaluated (Ito *et al.*, 2017; Li *et al.*, 2018; Wang *et al.*, 2019; Gao *et al.*, 2019; Wang *et al.*, 2020).

In *rin* mutant, almost all ripening-related phenotypes, including ethylene biosynthesis, carotenoid accumulation, fruit softening, and flavor synthesis, were significantly inhibited. In addition, the sepal size of *rin* mutant is increased, and the inflorescence is less ordered (Vrebalov *et al.*, 2002). Studies have shown that *rin* is formed by the deletion of the 3′ end of the *MADS-RIN* gene and the 5′ end of the *MADS-MC* gene, resulting in the formation of a *RIN–MC* fusion gene. *MADS-RIN* is considered to regulate tomato fruit ripening, while *MADS-MC* is considered to affect sepal development and inflorescence, and *rin* was considered a loss-of-function mutant of *RIN* (Vrebalov *et al.*, 2002). The phenotype of the *rin* mutant is a near complete inhibition of ripening, and based on this evidence, RIN was considered the core TF required for the tomato fruit ripening process, including ethylene biosynthesis and signal transduction, carotenoid synthesis, cell wall metabolism, aroma synthesis, sucrose metabolism, and other biological pathways (Qin et al., 2012, 2016; Fujisawa *et al.*, 2013; Wang *et al.*, 2014; Kumar *et al.*, 2016; Li *et al.*, 2017). Recent studies, however, have shown that the fusion protein RIN–MC in the *rin* mutant retains biological functions, and the role of RIN has been re-evaluated in light of this evidence (Ito *et al.*, 2017; Li *et al.*, 2018). The RIN protein segment of the RIN–MC fusion protein functions in binding DNA, while the adjacent MC region possesses a transcription repression function. This chimeric protein, RIN–MC, produced by the *rin* mutant is thus a gain-of-function mutant and active TF (Ito *et al.*, 2017; Li *et al.*, 2018) responsible for the inhibition of expression of ripening genes. It was concluded from this evidence that RIN was not required for the initiation of ripening but was essential for the completion of normal ripening (Ito *et al.*, 2017; Li et al., 2018, 2019). Compared with wild-type (WT), the *Cnr* mutant has reduced ethylene synthesis, fruit softening, and carotenoid synthesis in pericarp tissue (Eriksson *et al.*, 2004). Mapping and identification of *Cnr* by Manning *et al.* (2006) showed that *SPL-CNR* belongs to the SBP family of TFs. There was no alteration in the *SPL-CNR* DNA sequence, but its promoter region was hypermethylated, and the transcription of the *SPL-CNR* gene was inhibited, giving rise to the *Cnr* ripening mutant phenotype (Manning *et al.*, 2006). This was the first report of methylation affecting the expression of fruit ripening genes, but the exact cause of the methylation of *SPL-CNR* in the *Cnr* mutant remains unclear. Using CRISPR/Cas9 to edit *SPL-CNR* in WT fruit, Gao *et al.* (2019) found that the ripening of CR-CNR fruits was similar to that of WT tomatoes, and CR-CNR fruits fail to show a *Cnr* mutant phenotype. Therefore, the mechanism of action of the *Cnr* mutant and the function of *SPL-CNR* requires explanation and further study. Studies on the *nor* mutant and the function of NAC-NOR have lagged behind those of RIN and SPL*-*CNR and there is little information available regarding the mechanism of action of *nor* and the function of NAC-NOR. The synthesis of ethylene and carotenoids in the fruit of the *nor* mutant is significantly inhibited, and the fruit does not ripen. Giovannoni *et al*. (United States Patent, No. US 6 762 347 B1) discovered by map-based cloning that the *nor* mutant was caused by the deletion of two adenines in the third exon of the *NAC-NOR* gene, which belongs to the NAC gene family. Due to this frameshift mutation, the NAC*-*NOR protein in the *nor* mutant encodes a truncated NOR protein of 186 amino acids (aa) (NOR186), which disrupts the transcriptional activation region but preserves the complete DNA-binding region. Based on this evidence, the *nor* mutant phenotype was considered to be due to loss of function of the *NAC-NOR* gene, and NAC-NOR was considered to be a core TF regulating the initiation of tomato fruit ripening. Most NAC-NOR-related studies are based on the use of the *nor* mutant as experimental material. Yuan *et al.* (2016) compared the proteome differences between the *nor* mutant and WT tomato fruit by isobaric tags for relative and absolute quantification (iTRAQ) and found that the accumulation of many ripening-related and disease-resistance proteins was altered in the *nor* mutant. Additionally, the *NAC-NOR* mutation in Penjar tomato inhibited various metabolic processes and prolonged the shelf life of fruit (Kumar *et al.*, 2018), whereas the overexpression of *NAC-NOR* accelerated the senescence of tomato leaves (Ma *et al.*, 2019).

In addition to NAC-NOR, several other NAC TFs have been reported to be involved in regulating tomato fruit ripening. For example, the overexpression of *SlNAC1* in tomato resulted in a decrease in ethylene synthesis and the early softening of fruit, producing a yellow to orange phenotype (Ma *et al.*, 2014; Meng *et al.*, 2016). In addition, the silencing of *SlNAC4* in tomato fruit resulted in a 2–3 d delay in fruit ripening and significantly inhibited ethylene biosynthesis, chlorophyll degradation, and carotenoid accumulation (Zhu *et al.*, 2014). The ripening process in tomato fruit with CRISPR/Cas9 gene editing of *NOR-like1* (CR-NOR-like1) was significantly delayed for more than 2 weeks, and ethylene, carotenoid synthesis, and fruit softening were inhibited in CR-NOR-like1 fruit compared with WT (Gao *et al.*, 2018). Surprisingly, however, we have recently been unable to obtain a *nor* mutant phenotype in *NAC-NOR*-edited fruit using CRISPR/Cas9 (Gao *et al.*, 2019), which was published simultaneously by the de Maagd laboratory (Wang *et al.*, 2019), who demonstrated that NOR186 has an inhibitory function affecting ripening. Thus, the *nor* mutant may be a gain-of-function mutant, similar to *rin*, although the specific mechanism of action is unclear. If *nor* is a gain-of-function mutant, the role of *NAC-NOR* in the normal development and ripening of tomato and the function of the normal *NAC-NOR* gene in tomato fruit development and ripening need to be re-evaluated.

In this study, we investigated the results of CR-NOR and OE-NOR at the physiological, cellular, and molecular levels. The results showed that the residual protein NOR186 of the *nor* mutant could not only enter the nucleus but also bind to the promoters of NAC-NOR target genes, but could not activate them. While mixing the WT NOR protein and the *nor* mutation NOR186 protein, the activation effect of NOR target promoters was inhibited compared with the WT NOR protein present alone. In addition, overexpression of the *NAC-NOR* gene in the *nor* mutant did not restore the normal ripening phenotype of tomato, providing evidence for the gain-of-function of NOR186 in the *nor* mutant. Transcript accumulation studies indicate that NAC-NOR still plays an important role as a positive regulator in tomato fruit ripening. These results re-evaluated the role of NAC-NOR in tomato fruit ripening and help place it in the context of the transcriptional regulatory network regulating tomato fruit ripening.

## Materials and methods

### Plant materials and growth conditions

WT tomato (*Solanum lycopersicum*) cultivar Ailsa Craig (abbreviated as AC) and *NAC-NOR* gene transgenic lines were grown under controlled greenhouse condition with natural light. Standard greenhouse culture conditions with regular fertilizer application were used. Comparing the ripening process of tomato fruit by calculating the time from flowering to fruit discoloration, flowers were tagged at anthesis to record the ripening stages accurately through fruit development. Fruits of WT and *NAC-NOR* transgenic lines were harvested and collected at different ripening stages: mature green (MG), breaker (Br), and 3, 6, and 9 d after breaker (B+3, B+6, and B+9, respectively). Pericarp tissues of the harvested fruits were collected and frozen in liquid nitrogen immediately and stored at −80 °C until use.

### Total RNA isolation and quantitative real-time PCR analysis

RNA isolation from fruits or all other tissues was performed with the RNeasy Mini Kit (Qiagen, Germany) according to the manufacturer’s protocol. DNaseI (Qiagen, Germany) digestion was performed to remove genomic DNA. Transcript One-Step gDNA Removal and cDNA Synthesis SuperMix (TransGen, China) were used to synthesize cDNA from 2 μg total DNaseI-treated RNA. Quantitative real-time PCR (qRT-PCR) was conducted using SYBR Green PCR Master Mix (TransGen, China) with a CFX96 Real-Time PCR System (Bio-Rad, USA). The tomato *ACTIN* gene (Solyc03g078400) was used as the internal control. Relative gene expression values were calculated according to the 2^−△△Ct^ method (Livak & Schmittgen, 2001). For each sample, three biological replicates were included. All primers used for qRT-PCR are listed in Supplementary Table S1.

### Ethrel and 1-methylcyclopropene treatment

Wide-type tomato fruits at the mature green stage were treated with 0.4% ethrel or double distilled water (DDW) as control for 10 min (Breitel *et al.*, 2016), and then dried carefully and placed at room temperature for 12 h. Wild-type tomato fruits at the breaker stage were treated with 1.0 mg l^−1^ the ethylene signaling inhibitor 1-methylcyclopropene (1-MCP) or air as control for 16 h (Hao *et al.*, 2015). The pericarp tissues were sliced and frozen in liquid nitrogen after treatment for RNA isolation and qRT-PCR. For each treatment, three biological replicates from independent samples were included.

### Subcellular localization

The coding sequence (CDS) fragment of *NAC-NOR* and *NOR#19* without the stop codon were amplified respectively by PCR (primers used are listed in Supplementary Table S2) and then inserted into the pEAQ-GFP vector to produce the fusion construct NOR-GFP/NOR#19-GFP using the ClonExpress II One-Step Cloning Kit (Vazyme, China). The CDS region of *NOR186* and *RIN* (without the stop codon) was amplified respectively by PCR and inserted into the pEAQ-mCherry vector to produce the fusion protein NOR186–mCherry/RIN–mCherry using ClonExpress II One Step Cloning Kit (Vazyme, China). Then, pEAQ-NOR-GFP, pEAQ-NOR186-mCherry, pEAQ-NOR#19-GFP, pEAQ-RIN-mCherry, and the control vectors (pEAQ-GFP and pEAQ-mCherry) were transferred into *Agrobacterium tumefaciens* strain GV3101 and injected into 4-week-old tobacco leaves (Luo *et al.*, 2017). The green fluorescence of green fluorescent protein (GFP) and the red fluorescence of mCherry were observed and captured by a laser confocal microscope (Leica, Germany) after 48 h of infiltration.

### pYLCRISPR/Cas9Pubi-H-NOR and pCAMBIA-1300-221-NOR-3×HA vector construction and tomato genetic transformation

CRISPR-P (http://cbi.hzau.edu.cn/crispr/) was used to select four sgRNAs that targeted *NAC-NOR*; the four sgRNAs were cloned into the pYLCRISPR/Cas9Pubi-H binary plasmid using Golden Gate ligation (Ma *et al.*, 2015). The CDS sequence of *NAC-NOR* (without the stop codon) and a 3×HA-tag (at the C-terminus) were amplified respectively and cloned into the pCAMBIA-1300-221 vector to generate the 35S:NOR-3×HA construct for overexpression of the *NAC-NOR* gene (primers used are listed in Supplementary Table S2). The two vectors sequenced correctly were transformed into *A. tumefaciens* strain GV3101. Then pYLCRISPR/Cas9Pubi-H was transformed into AC tomato, while pCAMBIA-1300-221-NOR-3×HA was transformed into the WT tomato (AC) and the *nor* mutant as described previously (Van Eck *et al.*, 2006). The transgenic tomato lines were selected through their hygromycin resistance.

### DNA extraction and mutation analysis

Total genomic DNA was extracted from tomato leaves using the DNA Secure Plant Kit (Tiangen, China) according to the manufacturer’s instructions. The DNA was used as a template for amplifying the desired gene fragments using primers flanking the target sites or the possible off-target sites. For each target, two of the most likely off-target sites were tested. The oligonucleotide primers used for off-target analysis are listed in Supplementary Tables S3, S4.

### Gene overexpression analysis

Tomato fruits of the *NAC-NOR* gene overexpression in WT (OE-NOR in WT#2 and OE-NOR in WT#8) and in *nor* mutant (OE-NOR in *nor*#1 and OE-NOR in *nor*#16) at the stage of 20 d post-anthesis (dpa) were used for detection of the expression level of the *NAC-NOR* gene. Wild-type AC tomato fruits of 20 dpa were used as the control. Three biological replicates were performed.

### RNA sequencing and bioinformatics analysis

Total RNA was extracted from tomato fruits at the B+3 stage of WT, *nor*#11, OE-NOR in WT#2, and the *nor* mutant using the RNeasy Mini Kit (Qiagen, Germany); the genomic DNA was removed by DNase I (Qiagen, Germany) digestion. For each sample, three biological replicates were performed. mRNA enrichment, RNA-seq library construction, and sequencing were performed by Novogene (China). The clean data were mapped to the tomato reference genome (version SL2.50) using TopHat software (version 2.0.14). Fragments were assigned to genes by feature counts and count programs, and gene expression abundance was represented by the reads per kilobase of transcript per million mapped reads (RPKM) value. Differential expression analysis between *nor*#11 and WT, OE-NOR in WT#2 and WT, and *nor* mutant and WT were identified by DESeq2 Library (Anders and Huber 2010). The fold change of gene expression was calculated by RPKM_*nor*#11_/RPKM_WT_, RPKM_OE-NOR-in-WT#2_/RPKM_WT_, and RPKM_*nor*_/RPKM_WT_. Genes were considered as differentially expressed genes (DEGs) if |log_2_(fold change)|>1 and modified *P*_adj_<0.05. The common DEGs in *nor*#11/WT and OE-NOR in WT#2/WT with opposite regulatory patterns were screened for further analysis of the function of *NAC-NOR*.

### GO enrichment analysis

GO enrichment analysis was performed using the GO seq R package (Young *et al.*, 2010) based on DEGs that were common in *nor*#11/WT and OE-NOR in WT#2/WT with opposite regulatory patterns, with a threshold of *P*<0.05. Proteins were filtered based on their grouping into cellular components, molecular functions, and biological functions.

### Ethylene measurement

Tomato fruits of WT, *nor*#11, OE-NOR in WT#2, and *nor* mutant at different ripening stages (MG, Br, B+3, B+6, and B+9) were harvested, weighed, and placed at room temperature for 2 h to avoid measuring ‘wound ethylene’. Subsequently, each fruit was transferred into 300 ml gas-tight jars, tabbed, sealed, and incubated at room temperature. After 2 h, 1 ml gas samples were withdrawn immediately and then analysed by GC-2014 gas chromatography (Shimadzu, Japan). At least three biological replicates, each with three technical replicates, were performed for each sample. Ethylene concentrations were calculated by comparing sample peak areas with ethylene standards of known concentration, ethylene production (nl g^−1^ h^−1^) was calculated by the following formula: Ethylene production (nl g^−1^ h^−1^)=[*C*×(300−*M*)/*M*]/2, where *C* is ethylene concentration and *M* is the mass of the fruit.

### Carotenoid extraction and liquid chromatography–quadrupole time of flight mass spectrometry analysis

Carotenoids of tomato fruits at the B+9 stage from WT, *nor* mutant, *nor*#11, and OE-NOR in WT#2 were extracted as described previously (Fantini *et al.*, 2013) with minor modifications. The whole extraction process avoided light and was carried out at low temperature because carotenoids are volatile and oxidize in organic solvents. For each sample, four independent extractions were performed. Carotenoids were detected and identified, and the relative contents were determined by liquid chromatography–quadrupole time of flight mass spectrometry as previously described (Fantini *et al.*, 2013).

### Firmness measurement

In order to study the effect of NAC-NOR on tomato fruit softening, we measured the firmness of tomato fruits from WT, *nor* mutant, *nor*#11, and OE-NOR in WT#2 at different ripening stages (MG, Br, B+3, B+6, B+9) using a TA.XT Plus texture analyser (Stable Micro Systems, UK) with a 5 cm diameter cylindrical stainless probe. Fruit firmness examination was performed as described previously with minor revision (Wu and Abbott, 2002). The junction of outer and radial pericarp was compressed 2 mm at a test speed of 1 mm s^−1^; visible vascular bundles, fissures, and locular tissue were avoided. Each fruit was measured at two or three sites, and the average of the maximum force developed during the test was used as one biological replicate. At least three fruits per genotype and stage were measured.

### Protein expression and electrophoretic mobility shift assay

The partially coding sequence of *NAC-NOR* and the whole coding sequence of *NOR186* and *NOR#19* were amplified from the cDNA of AC, *nor* mutant, and *nor*#19, respectively. Then the products were inserted into the linearized pGEX-GST vector, which was digested with *Bam*HI and *Xho*I to produce recombinant NOR/NOR186/NOR#19 protein with a glutathione *S*-transferase (GST) tag using ClonExpress II One Step Cloning Kit (Vazyme, China). The N-GST– NOR/NOR186/NOR#19 fusion protein was expressed in *Escherichia coli* strain Rosetta (DE3) and induced with 1 mM isopropyl β-D-1-thiogalactopyranoside at 37 °C for 6 h, at 37 °C for 6 h, and at 20 °C for 12 h, respectively, and further purified with Glutathione Sepharose 4B (GE Healthcare, Sweden) following the manufacturer’s protocol. The oligonucleotide probes (shown in Supplementary Table S5) containing the NAC recognize sequence (NACRS) [TGA][ACG]CGT[GA][TA] (O’Malley *et al.*, 2016) regions derived from the promoters of *SlACS2*, *SlGgpps2*, and *SlPL* were synthesized (Sangon Biotech, China) and biotin-labeled using the Biotin 3′ End DNA Labeling Kit (Thermo Scientific, USA). An electrophoretic mobility shift assay (EMSA) was carried out as previously described (Han *et al.*, 2016; Tan *et al.*, 2018). Both the unlabeled probes and the mutant probes were used for competition; GST protein alone was used as negative control.

### Dual-luciferase reporter assay

A dual-luciferase reporter assay was performed to analyse the transcriptional activity of NAC-NOR, NOR186, and NOR#19 with the promoters of *SlACS2*, *SlGgpps2*, and *SlPL*. The CDS fragment of NAC-NOR, NOR186, and NOR#19 was amplified and cloned into the 35S promoter-driven pEAQ vector (Sainsbury *et al.*, 2009) to construct the effector vectors pEAQ-NOR, pEAQ-NOR186, and pEAQ-NOR#19. The 1–2 kb promoter regions of *SlACS2*, *SlGgpps2*, and *SlPL* that were reported in our previous study (Gao *et al.*, 2018) were amplified and inserted into the linearized double-reporter vector pGreenII 0800-LUC (Hellens *et al.*, 2005) to construct pGreenII 0800-SlACS2-LUC/pGreenII 0800-SlGgpps2-LUC/pGreenII 0800-SlPL-LUC using ClonExpress II One Step Cloning Kit (Vazyme, China). Subsequently, the recombinant effector was transferred into *A. tumefaciens* strain EHA105, and the reporters were transferred into EHA105 (pSoup). The *A. tumefaciens* containing effector/reporter was inoculated, collected, and suspended in infiltration buffer (10 mM MES, 10 mM MgCl2, 150 mM acetosyringone). The effector and reporter were mixed (9:1) and then co-infiltrated into 4-week-old tobacco (*Nicotiana benthamiana*) leaves as described previously (Fan *et al.*, 2016; Tan *et al.*, 2018). To investigate whether NOR186 has a competitive effect on the NOR protein, NOR186 was added as the same concentration with NOR, and then mixed with the reporter at 9:1 to perform the infiltration. After 48 h of infiltration, LUC and REN luciferase activities were measured respectively using the dual-luciferase assay kit (Promega, USA) with the Luminoskan Ascent Microplate Luminometer (Thermo Fisher Scientific, USA). The results were calculated as the ratio of LUC to REN. At least five biological replicates were performed for each combination.

### Statistical analysis

Significance analysis of corresponding experimental data was conducted using IBM SPSS Statistics 20 software. Pairwise comparisons were computed using Student’s *t*-test (*P<0.05 and **P<0.01), while multiple comparisons were subjected to ANOVA using Duncan’s test. Statistically significant differences (*P*<0.05) are indicated by different lowercase letters.

## Results

### The expression of *NAC-NOR* is ripening-related and induced by ethylene

**Fig. 1.**
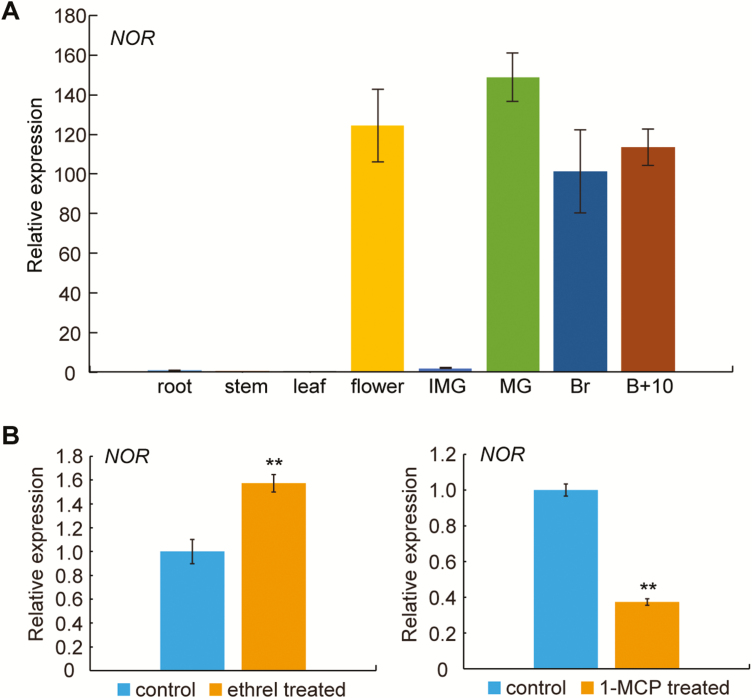
The accumulation pattern of *NAC-NOR* gene transcripts in different tomato organs, during tomato fruit ripening, and in response to ethylene and 1-MCP. (A) The accumulation pattern of *NAC-NOR* gene transcripts in different tomato plant organs (root, stem, leaf, and fruit) at different stages. B+10, 10 d after breaker; Br, breaker; IMG, immature green; MG, mature green. The *ACTIN* gene was used as the internal control. Bars represent ±SD of three independent replicates. (B) Accumulation of the *NAC-NOR* gene transcripts in WT fruit after treatment with ethrel or 1-MCP. WT tomato fruits at the mature green stage and breaker stage were used for ethrel treatment (10 min at a concentration of 0.4% ethrel) and 1-MCP treatment (16 h at a concentration of 1.0 mg l^−1^), respectively. *ACTIN* gene was used as the internal control. Error bars indicate ±SD of three biological replicates. Asterisks indicate significant differences determined by Student’s *t*-test (**P<0.01).

To investigate the activity of the *NAC-NOR* gene during fruit development, the accumulation of *NAC-NOR* transcripts in various WT tomato plant organs and during fruit development and ripening was measured by qRT-PCR. The results showed that the expression of the *NAC-NOR* gene in vegetative organs such as root, stem, and leaf of tomato was low, while it was high in reproductive organs such as flower and fruit (Fig. 1A), which suggested that it may play an important role in tomato fruit ripening. Ethylene is a key hormone in the ripening of climacteric tomato, and many ripening-related genes are induced by ethylene during fruit ripening (Barry and Giovannoni, 2007; Kumar *et al.*, 2014). To study the relationship between *NAC-NOR* expression and ethylene, we used treatment with an ethylene-generating compound (ethrel) and an ethylene perception inhibitor (1-methylcyclopropene; 1-MCP) to treat WT tomato fruits at mature green and breaker stages of fruit ripening, respectively. The results showed that the expression of the *NAC-NOR* gene in tomato fruit was induced by ethylene but inhibited by 1-MCP (Fig. 1B). Based on the above results, it could be hypothesized that the *NAC-NOR* gene is specifically expressed during tomato ripening and induced by exogenous ethylene treatment. This is consistent with a role for *NAC-NOR* in tomato fruit ripening, but this hypothesis requires further functional verification.

### CRISPR/Cas9-mediated mutagenesis of the *NAC-NOR* gene inhibited fruit ripening but resulted in a phenotype that was different from that of the *nor* mutant

**Fig. 2.**
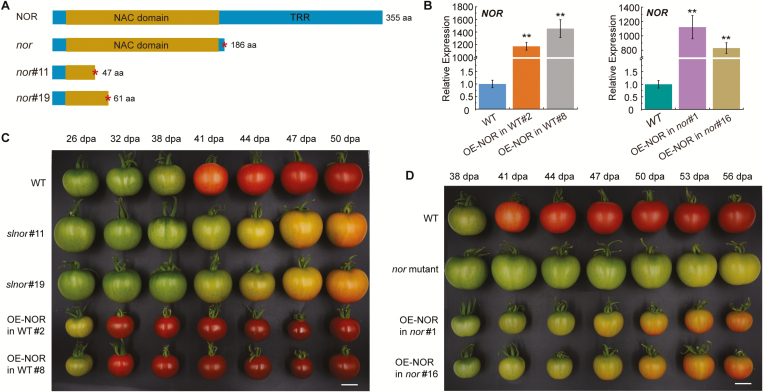
The phenotypes of CR-NOR and OE-NOR tomato fruit. (A) The structure of the NAC-NOR protein in the WT, *nor* mutant, and CR-NOR tomato plants. WT tomatoes encode a full-length NAC-NOR protein of 355 aa. The *nor* mutant produces a truncated protein of 186 aa (NOR186). *nor*#11 produces a truncated protein of 47 aa, and *nor*#19 produces a truncated protein of 61 aa. The NAC domain is present in NOR186 but is absent in *nor*#11 and *nor*#19. (B) The expression level of *NOR* gene in fruit of OE-NOR lines in WT (left) and the fruit of OE-NOR lines in the *nor* mutant background (right). Error bars represent ±SD of three biological replicates. **P<0.01 (Student’s *t*-test). (C) The phenotypes of CR-NOR and OE-NOR fruits in the WT background. (D) The phenotype of OE-NOR fruits in the *nor* background. dpa: d post-anthesis; scale bar: 2 cm (C, D).

To further study the role of NAC-NOR in tomato fruit ripening, we carried out CRISPR/Cas9-mediated mutagenesis of the *NAC-NOR* gene by transgenic methods. Two homozygous CR-NOR plants named *nor*#11 and *nor*#19 were obtained, which encode truncated NAC-NOR proteins with 47 and 61 aa, respectively (Fig. 2A; Gao *et al.*, 2019). The DNA-binding and transcriptional regulatory region (TRR) of the NAC-NOR protein were both destroyed in *nor*#11 and *nor*#19 (Fig. 2A). These results suggest that *nor*#11 and *nor*#19 are loss-of-function mutants suitable for the analysis of *NAC-NOR* gene function. The CR-NOR phenotype is not consistent with the mature phenotype of the *nor* mutant (Wang *et al.*, 2019; Gao *et al.*, 2019); however, the authors of these studies did not provide sufficient information to describe the ripening process of CR-NOR fruit. To study the effect of NAC-NOR on the fruit development and ripening process in tomato, the days from flowering to fruit color break were recorded. Color break in *nor#11* and *nor#19* fruits was delayed only 3 d compared with that in WT fruits, which is accordance with the CRISPR-NOR phenotype described by Wang *et al.* (2019), but the subsequent accumulation of carotenoids in *nor*#11 and *nor*#19 fruits was significantly reduced compared with that in the control tomato fruit (Fig. 2D). The WT fruit turned completely red 9 d after breaker (B+9), while the fruits of *nor*#11 and *nor*#19 maintained an orange-yellow phenotype. The breaker stage of the *nor* mutant occurred approximately 50 d after anthesis, significantly later than that of CR-NOR, and the accumulation of pigments in the fruit of the *nor* mutant was significantly inhibited compared with that of WT (Fig. 2D). Even 30 d after color break, the fruit of the *nor* mutant still remained light yellow, while the color of *nor#*11 and *nor#*19 fruits was orange-red and more similar to that of the WT fruit (Gao *et al.*, 2019). The color of CR-NOR fruit was obviously different from that of the *nor* mutant fruit at the final ripening stage (B+30) (Gao *et al.*, 2019). These results indicated that CR-NOR inhibited tomato fruit ripening, but this inhibition was much less severe than the inhibition of fruit ripening in the *nor* mutant fruit; these results confirmed Wang *et al*.’s results and our earlier observations (Gao *et al.* 2019; Wang *et al.*, 2019) and provide new evidence to explain them.

### Overexpression of *NAC-NOR* significantly accelerates the fruit ripening process in tomato but cannot completely restore the ripening phenotype of the *nor* mutant fruit

To provide additional biological evidence for the role of the *NAC-NOR* gene in fruit ripening, we overexpressed *NAC-NOR* in WT tomato. A total of 10 T_0_ transgenic tomatoes were obtained. Two lines with elevated *NAC*-*NOR* gene expression were selected as OE-NOR in WT#2 and OE-NOR in WT#8 for further research. The expression of *NAC-NOR* in OE-NOR in WT#2 and OE-NOR in WT#8 was higher than that in WT tomato fruits (Fig. 2B). At the same time, we observed and recorded the ripening characteristics of OE-NOR fruits. The results showed that color break in WT tomatoes occurred 38 d after flowering, but in OE-NOR in WT#2 and OE-NOR in WT#8, color break occurred approximately 26 d after flowering (Fig. 2C). We also found that OE-NOR in WT#2 and OE-NOR in WT#8 fruits accumulated pigments more rapidly after color break and finally showed a deep red phenotype (Fig. 2C). We also overexpressed the *NAC-NOR* gene in the *nor* mutant (Fig. 2B). The results showed that although OE-NOR in the *nor* mutant significantly accelerated fruit ripening compared with the *nor* mutant, it could not completely restore the ripening phenotype of the *nor* mutant to the WT pattern (Fig. 2D). Color break in the *nor* mutant with OE-NOR occurred earlier than that in *nor* mutant fruit, but later than that in WT (Fig. 2D). These results indicate that the phenotype of the *nor* mutant is not explained solely by the loss function of *NAC-NOR*. OE-NOR in WT tomato fruit, however, accelerated tomato fruit ripening, implying that NAC-NOR does play an important role in regulating the tomato fruit development and ripening process.

### NAC-NOR regulates several aspects of tomato fruit ripening

**Fig. 3.**
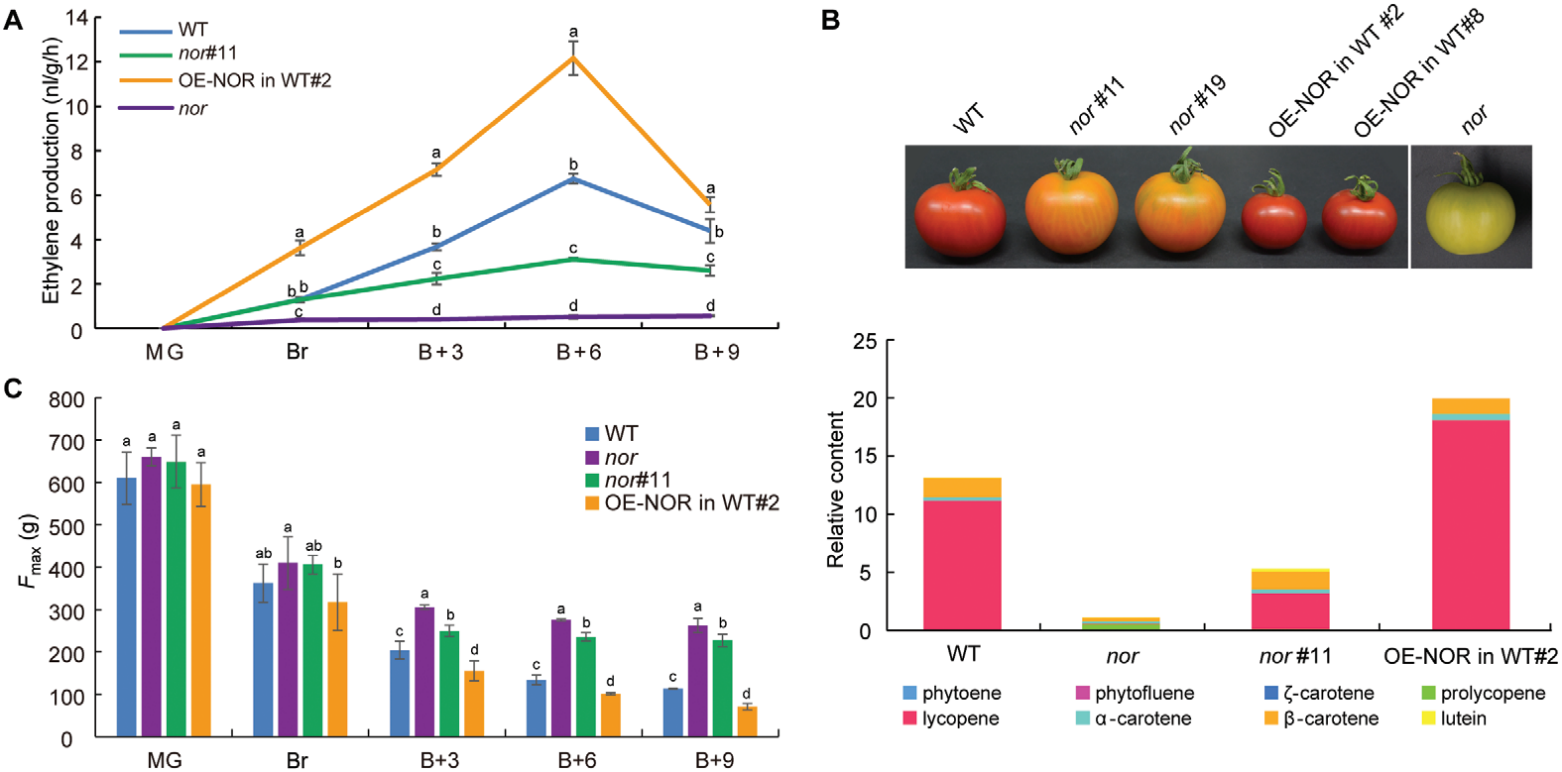
NAC-NOR regulates many aspects of tomato fruit development and ripening, including ethylene production, carotenoid content, and fruit firmness. (A) Ethylene production. Fruits were harvested at different stages, and placed at room temperature for 2 h to allow ‘wound ethylene’ to subside. Subsequently, each fruit was transferred into 300 ml gas-tight jars, tabbed, sealed, and incubated at room temperature. After 2 h, 1 ml gas samples were withdrawn immediately and then analysed; significant differences (*P*<0.05) are indicated by lowercase letters. (B) Carotenoid content of the fruit of four different samples (WT, *nor*, and *nor*#11 and OE-NOR in WT#2) was quantified by HPLC at the B+9 stage. The upper panel shows the phenotype of tomato fruits at the B+9 stage, and the lower panel shows the carotenoid content. Significant differences (*P*<0.05) are indicated by lowercase letters. (C) The firmness of tomato fruits. Fruits were harvested at different stages, kept at room temperate, and used for each measurement of the force required for probe penetration (*F*_max_). Significant differences (*P*<0.05) are indicated by lowercase letters. B+3, 3 d after breaker; B+6, 6 d after breaker; B+9, 9 d after breaker; Br, breaker; MG, mature green.

To study the effects of *NAC-NOR* on the important physiological characteristics of tomato fruit ripening, we measured ethylene production, carotenoid content, and fruit firmness of WT, *nor*, *nor*#11 and OE-NOR in WT#2 tomato fruits at different ripening stages (MG, Br, B+3, B+6, and B+9). The results showed that ethylene production (Fig. 3A), carotenoid content (Fig. 3B), and fruit softening (Fig. 3C) of the *nor*#11 fruit were significantly reduced compared with those of WT fruit at the same stage; ethylene production (Fig. 3A), carotenoid content (Fig. 3B), and fruit softening (Fig. 3C) of the OE-NOR in WT#2 fruit were significantly higher than those of the WT control fruit at the same stage, whereas the *nor* mutant fruit produced a very small amount of ethylene (Fig. 3A) and carotenoids (Fig. 3B), and the fruit firmness (Fig. 3C) of *nor* mutant was significantly higher than that of WT and CR-NOR mutants from 3 d after the breaker (B+3) stage. These results indicated that the *NAC-NOR* gene was a positive regulator of tomato fruit ripening but the inhibition of ripening in the *nor* mutant was much more severe than that in CR-NOR mutants.

**Fig. 4.**
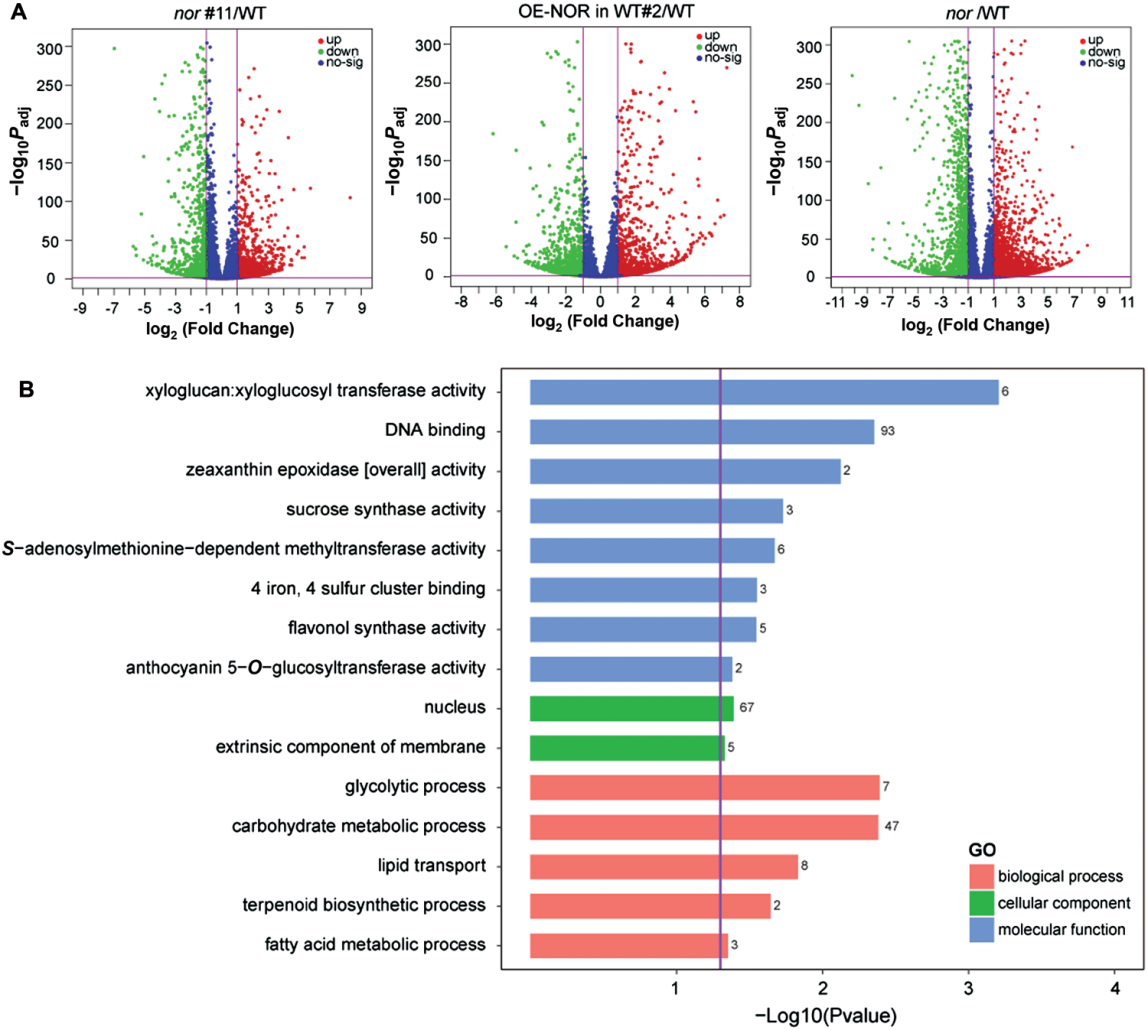
RNA-seq and GO analysis of fruit from the WT, OE-NOR, CR-NOR, and *nor* mutant plants. (A) RNA-seq data of *nor*#11, OE-NOR#2 in WT, *nor*, and WT visualized by volcano plots. Each point represents a DEG. Red points represent up-regulated genes, and green points represent down-regulated genes (*nor*#11/WT or OE-NOR#2 in WT/WT or *nor*/WT). |Log_2_(fold change)|>1 and *P*_adj_=0.05 are marked with purple lines. (B) GO functional enrichment analysis of DEGs between *nor*#11 and WT. *P*=0.05 is marked with a purple line, and the gene numbers enriched in each category are indicated to the right of the colored bars.

**Fig. 5.**
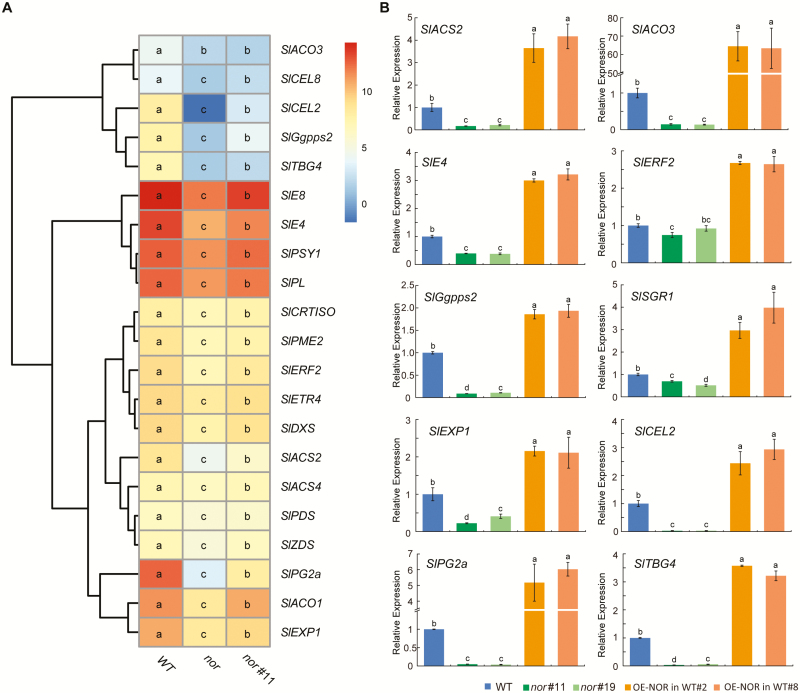
NAC-NOR regulates the expression of genes related to tomato fruit ripening. (A) Heatmap of key genes in ethylene biosynthesis and signal transduction pathways, carotenoid accumulation, and fruit softening of WT, *nor*#11, and *nor*. Log_2_RPKM was used to normalize the RNA-Seq data in the heatmap, and the significant differences are shown in each column by lowercase letters. (B) Validation of RNA-seq results by qRT-PCR. Ten genes with different expression levels were selected to be tested by qRT-PCR in *nor*#11, *nor*#19, OE-NOR in WT#2, and OE-NOR in WT#8 and WT fruits at the B+3 stage. The *ACTIN* gene was used as the internal control. Bars represent ±SD of three independent replicates.

To further elucidate the molecular mechanism of NAC-NOR regulation of tomato fruit ripening, we performed transcriptome sequencing (RNA-sequencing) on tomato fruits of *nor*#11, OE-NOR in WT#2, *nor* mutant, and WT at 3 d after color break. We screened DEGs based on the threshold of |log_2_(fold change)|>1 and *P*_adj_<0.05. When all data were compared with the transcript accumulation in the WT fruits, the results showed that 1428 genes were significantly up-regulated and 997 genes were significantly down-regulated in *nor*#11. A total of 1185 genes were significantly up-regulated and 1406 genes were significantly down-regulated in OE-NOR in WT#2, while 2673 genes were up-regulated and 1827 genes were down-regulated in the *nor* mutant (Fig. 4A). We screened the key DEGs related to fruit ripening from *nor*#11 and compared the transcript levels to those in OE-NOR in WT#2 (Table 1). GO enrichment analysis of DEGs by GO seq showed that CR-NOR affected not only carotenoid biosynthesis and cell wall metabolism (zeaxanthin epoxidase activity, terpenoid biosynthesis process, xyloglucan: xyloglucosyl transferase activity) but also the synthesis or metabolic processes of sugar, carbohydrate, fatty acid, anthocyanin, flavanol, and other substances (sucrose synthase activity, *S*-adenosylmethionine-dependent methyltransferase activity, flavonol synthase activity, anthocyanin 5-*O*-glucosyltransferase activity, glycolysis process, carbohydrate metabolic process, lipid transport, fatty acid metabolic process) (Fig. 4B). Combining the DEG data from the RNA-seq data of the above materials, we found that the extent of down-regulation of genes related to ethylene synthesis and signal transduction (*SlACS2*, *SlACS4*, *SlACO1*, *SlACO3*, *SlETR4*, *SlE4*, *SlE8*, and *SlERF2*), carotenoid metabolism (*SlDXS*, *SlGgpps2*, *SlPSY1*, *SlPDS*, *SlZDS*, and *SlCRTISO*), and fruit softening (*SlPG2a*, *SlPME2*, *SlTBG4*, *SlPL*, *SlCEL2*, *SlCEL8*, and *SlEXP1*) in the *nor* mutant was significantly higher than that of the CR-NOR mutant. Different transcriptional levels of these key ripening-related genes may be one reason for the differences in phenotype between the *nor* mutant and CR-NOR mutant (Fig. 5A). To verify the validity of the sequencing results as well as to confirm the regulatory effect of NAC-NOR on the key ripening-related genes, qRT-PCR was used to measure the expression of selected genes (*SlACS2*, *SlACO3*, *SlE4*, *SlERF2*, *SlGgpps2*, *SlSGR1*, *SlPG2a*, *SlTBG4*, *SlEXP1*, and *SlCEL2*) at the B+3 stage of WT, *nor*#11, *nor*#19, OE-NOR in WT#2, and OE-NOR in WT#8 (Fig. 5B), and the results were consistent with the sequencing results. Taken together, the above results indicated that NAC-NOR regulates several aspects of tomato fruit ripening and is beyond any doubt important, but the phenotype of the *nor* mutant is not only caused by the loss of NAC-NOR function.

**Table 1.** Genes involved in tomato fruit ripening regulated by NAC-NOR

Gene name	Gene ID	Log_2_(fold change) *nor*#11/WT	Log_2_(fold change) OE-NOR in WT#2/WT	Annotation
*ACS2*	Solyc01g095080	−2.90	0.66	1-Aminocyclopropane-1-carboxylate synthase
*ACO3*	Solyc07g049550	−2.69	4.32	1-Aminocyclopropane-1-carboxylate oxidase
*E4*	Solyc03g111720	−1.95	0.50	Peptide methionine sulfoxide reductase msrA
*ERF2*	Solyc09g075420	−1.10	0.40	Ethylene responsive transcription factor
*Ggpps2*	Solyc04g079960	−3.91	−0.09	Geranylgeranyl pyrophosphate synthase
*Lcy-b*	Solyc04g040190	1.35	−0.60	Lycopene β-cyclase
*SGR1*	Solyc08g080090	−1.17	0.53	Senescence-inducible chloroplast stay-green protein
*PG2a*	Solyc10g080210	−4.79	1.42	Polygalacturonase
*PME2*	Solyc07g064180	−1.40	−0.20	Pectinesterase
*TBG4*	Solyc12g008840	−5.40	0.73	β-Galactosidase
*EXP1*	Solyc06g051800	−1.80	0.33	Expansin
*CEL2*	Solyc09g010210	−5.34	0.38	Endoglucanase
*CEL8*	Solyc08g082250	−1.85	0.72	Endoglucanase
*TAGL1*	Solyc07g055920	−1.21	0.49	Agamous MADS-box transcription factor

### The *nor* mutant truncated protein NOR186 is located in the nucleus and can bind to but not activate the promoters of ripening-related target genes

**Fig. 6.**
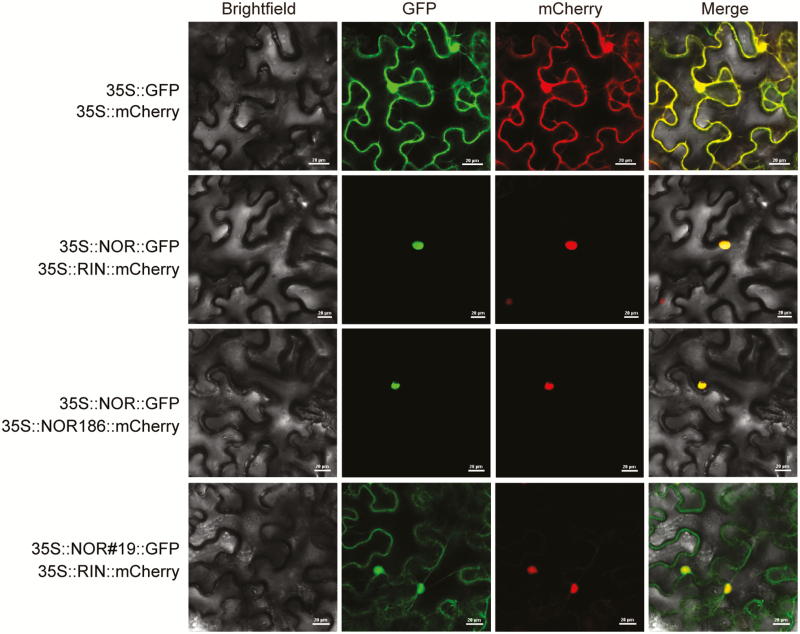
The truncated NOR186 protein is located in the nucleus. Subcellular localization of NAC-NOR, NOR186 (residual NOR truncated protein in the *nor* mutant), and NOR#19 (NOR truncated protein in NOR CRISPR line *nor*#19) was performed in 4-week-old tobacco (*N. benthamiana*) leaves and photographs were taken 48 h post-incubation under confocal microscopy. GFP and mCherry were co-expressed as the control. NOR–GFP and RIN–mCherry (marker protein for the nucleus) were co-expressed in the nucleus of tobacco leaves, NOR186–mCherry and NOR–GFP were also co-expressed in the nucleus, while NOR#19–GFP and the GFP protein alone were dispersed throughout the cell. Green and red fluorescence images were taken in a dark field, while the outline of the cell was photographed in a brightfield. Scale bars: 25 μm.

**Fig. 7.**
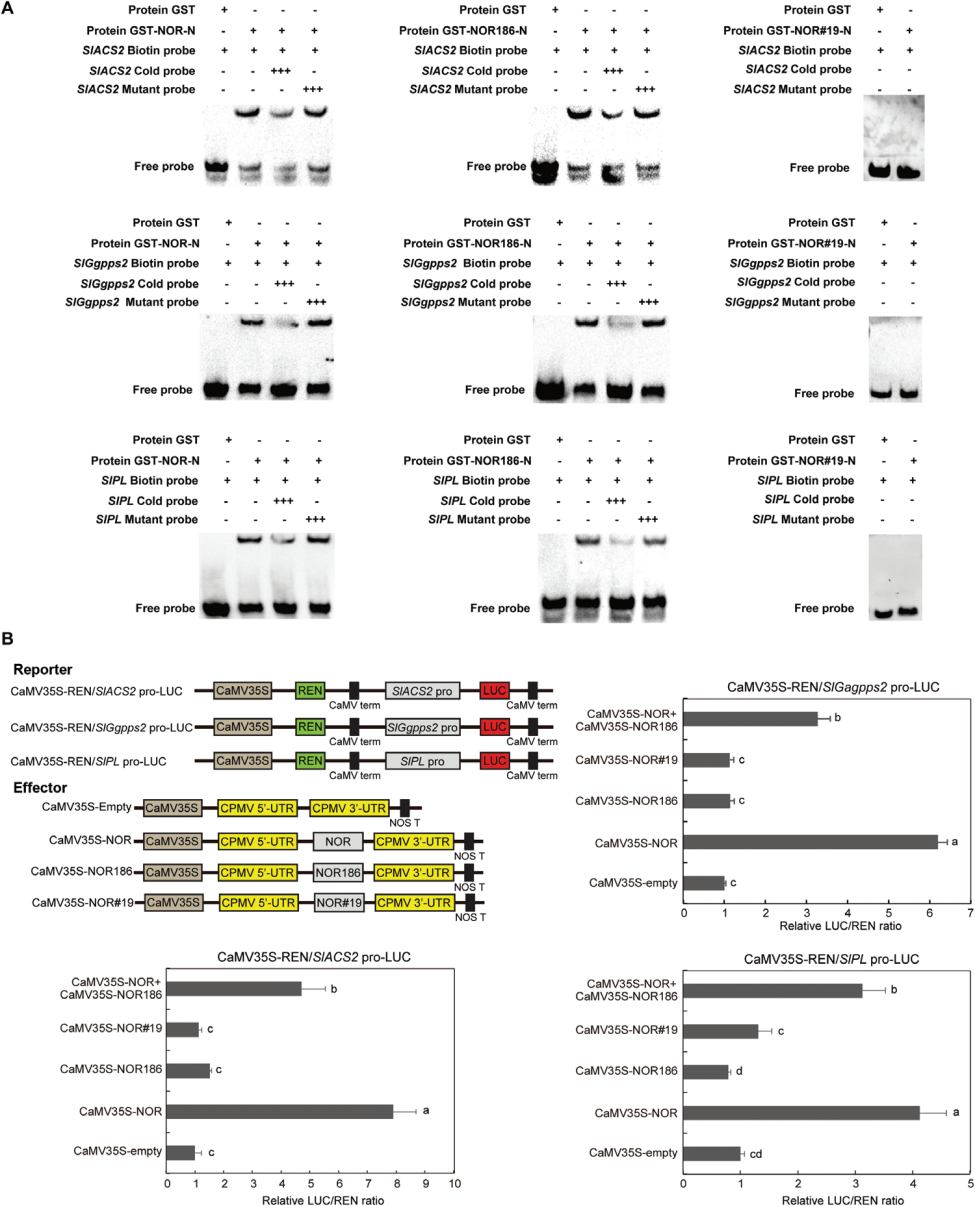
The truncated NOR186 protein can bind to but not activate the promoters of ripening-related target genes, including *SlACS2*, *SlGgpps2*, and *SlPL*. (A) EMSA of *in vitro* binding of NOR, truncated NOR protein (NOR186) and truncated CRISPR-NOR protein NOR#19 to the promoters of target genes *SlACS2*, *SlGgpps2*, and *SlPL*. Purified GST-tagged NOR/NOR186/NOR#19 protein was incubated with the biotin-labeled WT probe containing NACRS. Competition for NOR/NOR186 binding was performed with 1000× cold probes containing the WT NACRS or mutated NACRS. The DNA–protein complexes were separated on native polyacrylamide gels. The symbols ‘+’ and ‘–’ represent presence and absence, respectively, and ‘+++’ indicates increasing amounts. (B) Transient expression assay for NOR/NOR186/NOR#19/NOR+NOR186 activation of the promoters of *SlACS2*, *SlGgpps2*, and *SlPL*. The reporter and effector vectors in each experiment (such as CaMV35S-REN/SlACS2-pro-LUC with CaMV35S-NOR) were co-introduced into tobacco (*N. benthamiana*) leaves by *Agrobacterium* GV3101. The activation of *SlACS2/SlGgpps2/SlPL* promoter by NOR/NOR186/NOR#19/NOR+NOR186 was shown by the ratio of LUC to REN. Each value represents the means of five or six biological replicates. Different lowercase letter represent a significant difference (Duncan’s multiple range test, *P*<0.05).

The *nor* mutant gene lacks two adenines, resulting in the early termination of translation and the production of the truncated 186-aa protein NOR186. Despite the lack of a TRR, the complete DNA-binding region is present in NOR186 (Fig. 2A). Subcellular localization showed that NOR186 was located in the nucleus, while the NOR#19 truncated protein from *nor*#19, generated by CRISPR/Cas9, was not located in the nucleus (Fig. 6), which indicated that *nor*#19 is a true loss-of-function mutant. Promoter analysis and RNA-seq analysis indicated that *SlACS2*, *SlGgpps2*, and *SlPL*, which are involved in ethylene biosynthesis, carotenoid synthesis, and fruit softening, respectively, are the target genes of the NAC-NOR protein. EMSA results showed that the NOR186 protein could bind to the conserved promoter regions of the three target genes, while the truncated protein NOR#19 could not bind to these promoter regions (Fig. 7A). Dual-luciferase reporter assay results showed that the relative LUC/REN ratio in tobacco leaves co-transformed with CaMV35S-NOR and CaMV35S-REN/pSlACS2-LUC, CaMV35S-REN/pSlGgpps2-LUC, or CaMV35S-REN/pSlPL-LUC was signiﬁcantly higher than when co-transformed with CaMV35S-Empty and CaMV35S-REN/pSlACS2-LUC, CaMV35S-REN/pSlGgpps2-LUC, or CaMV35S-REN/pSlPL-LUC, but there was no significant difference between NOR186/NOR#19 and the control group. These results indicated that NAC-NOR could activate the promoter activity of *SlACS2*, *SlGgpps2*, and *SlPL* in tobacco, while NOR186 and NOR#19 could not (Fig. 7B). When CaMV35S-NOR was co-expressed with CaMV35S-NOR186 (mixing the WT NOR protein and the *nor* mutation NOR186 protein), the activation effect of *SlACS2*, *SlGgpps2*, and *SlPL* promoters was inhibited compared with the WT NOR protein occurring alone. This result demonstrated that NOR186 is a dominant-negative protein, which is consistent with the hypothesis of Wang *et al.* (2019). These results also explain why overexpressed NOR in the *nor* mutant cannot fully restore its immature phenotype. Based on the above results, it is concluded that NAC-NOR protein can enter the nucleus, bind to and activate the promoters of its target genes, while NOR186 also can enter the nucleus and bind to the promoter region of the target genes but cannot activate their promoters and play the role of a dominant-negative protein. Thus, *nor* is a gain-of-function mutant.

## Discussion

### *NAC-NOR* gene editing delays the initiation of tomato fruit ripening

Mature fruit of *nor* mutant produces no ethylene burst, undergoes very little change in carotenoid content, and almost completely fails to ripen (Mizrahi *et al.*, 1976; Davies *et al.*, 1991; White 2002; Kumar *et al.*, 2018). Based on these results, NAC-NOR has long been considered one of the core TFs regulating the initiation and progression of tomato fruit ripening (Giovannoni, 2004, 2007). However, the fruit of homozygotes with *NAC-NOR* gene mutations induced by CRISPR/Cas9 technology shows a much less severe phenotype than the *nor* mutant, and the fruit undergoes significant ripening (Fig. 2C, D; Gao *et al.*, 2019; Wang *et al.*, 2019). The ripening of CR-NOR tomato fruit was partially inhibited, the breaker stage of CR-NOR fruit was delayed by only 3 d compared with that of WT fruit (Fig. 2C). Ethylene and carotenoids were still synthesized, and fruit softening was initiated in CR-NOR fruit (Fig. 3). The color of CR-NOR tomato fruit was close to that of WT fruit 30 d after the color break stage, but the mature phenotype was obviously different (Gao *et al.*, 2019). From these observations, it can be inferred that *NAC-NOR* gene editing delays the initiation of tomato fruit ripening. Recent studies have found that the RIN–MC fusion protein of the *rin* mutant, rather than being a loss of function mutation, is a gain-of-function TF that regulates tomato fruit ripening by transcriptional inhibition (Ito *et al.*, 2017; Li *et al.*, 2018). This new evidence shows that the *RIN* gene is not a necessary element for ripening initiation but is required for the development of full ripening attributes (Ito *et al.*, 2017; Li *et al.*, 2018). The question arises as to whether there is a similar explanation for the mechanism of action of the *nor* mutation.

### NOR186 protein from the *nor* mutant inhibits accumulation of some ripening genes and *nor* is a gain-of-function mutation

**Fig. 8.**
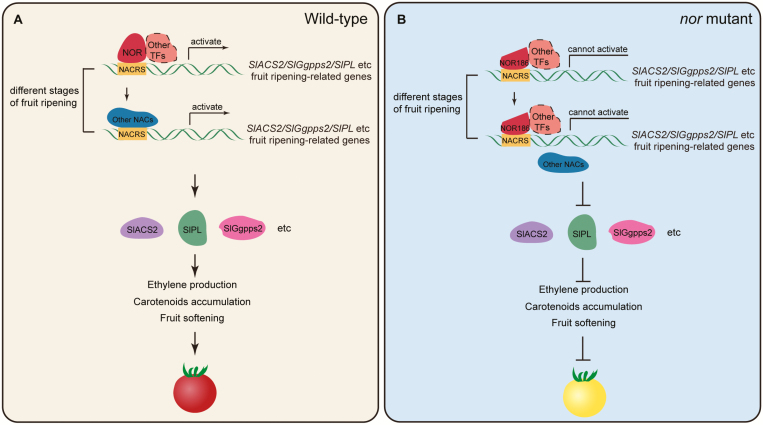
Model of NAC-NOR and the mutant NOR186 in regulation of tomato fruit ripening. (A) In the WT tomato, the complete NAC-NOR protein can enter the nucleus, interact with other ripening-related TFs, bind to the NACRS (which other NAC TFs also can bind to at different ripening stages) of ripening-related genes, such as *SlACS2*, *SlGgpps2*, and *SlPL*, and activate their expression, promoting ethylene production, carotenoid accumulation, and fruit softening. (B) In the *nor* mutant, the truncated NOR186 protein can enter the nucleus, interact with other ripening-related TFs, and bind to the NACRS of ripening-related genes, such as *SlACS2*, *SlGgpps2*, and *SlPL*, but cannot activate the transcription of these genes due to the lack of a transcriptional regulation region. On the other hand, the complex of NOR186–other TFs may occupy the NACRS promoter regions of its target genes when other NAC TFs need to bind at the following ripening stages, thereby inhibiting the binding of other NAC TFs to these regions. As a result, ethylene production, carotenoid accumulation, and fruit softening are depressed.

The de Maagd laboratory at Wageningen University and our laboratory generated CR-NOR mutants in WT tomato by CRISPR/Cas9 technology and showed that the ripening of CR-NOR tomato fruit was partially inhibited (Gao *et al.*, 2019; Wang *et al.*, 2019). The color change in CR-NOR fruit after color break was slower than that in WT fruit, and CR-NOR fruit became orange, rather than red, 9 d after color break, which is obviously different from the almost completely inhibited ripening phenotype of *nor* mutant fruit (Fig. 2C; Wang *et al.*, 2019). Wang *et al.* (2019) further edited NOR186 in the *nor* mutant to obtain a phenotype similar to that of CR-NOR in the WT background. They concluded that the mutant *nor* protein (here referred to as NOR186) is a dominant-negative protein. They also speculated that the truncated protein (NOR186) in the *nor* mutant still has the ability to interact with other NAC proteins and to bind DNA without transcriptionally activating its targets (Wang *et al.*, 2019). Here, we provide further experimental evidence for the hypothesis that NOR186 is localized in the nucleus and is capable of binding to the promoters of *SlACS2*, *SlPL*, and *SlGgpps2* target genes, but is incapable of activating them (Fig. 7). This contrasts with the behavior of NOR#19, produced by CRISPR-Cas9, which does not enter the nucleus, does not bind to and cannot activate the promoters of *SlACS2*, *SlPL*, and *SlGgpps2* (Fig. 7). Furthermore, overexpression of *NAC-NOR* in the *nor* background could not completely restore the ripening quality attributes to the level of WT fruits, as exemplified by their inability to turn completely red 15 d after color break (Fig. 2D). This phenotype was explained by co-expressing CaMV35S-NOR with CaMV35S-NOR186; the activation effect of *SlACS2*, *SlGgpps2*, and *SlPL* promoters was inhibited compared with the WT NOR protein present alone (Fig. 7B). Based on our above results, and combining the hypothesis of Wang *et al*., we constructed a model of the NOR and *nor* mutants’ functions in tomato fruit (Fig. 8; Wang et al., 2019, 2020). In WT tomato, WT NOR protein interacts with other fruit ripening-associated TFs including other NAC TFs, binds to the NACRS, and activates ripening-associated target genes such as *SlACS2*, *SlPL*, and *SlGgpps2* to regulate tomato fruit ripening. Besides, other NAC TFs that are not interact with NOR protein can also bind to the NACRS and activate the same genes at the following tomato ripening stages (Fig. 8A). In *nor* mutant fruit, NOR186 lacks the TRR, but retains the DNA binding region, and the protein complex of NOR is still present and can bind to the promoters of the target genes, but is unable to activate them. In addition, NOR186 can play a space-occupying role and stop other NAC TFs from binding the same NACRS site of the same target genes such as *SlACS2*, *SlPL*, and *SlGgpps2* at the following tomato ripening stages (Fig. 8B). However, the specific mechanism of *nor* functional transformation in the mutants is unclear, and needs further research.

### NAC-NOR is a positive regulator of tomato fruit ripening, and its function is different from that of NOR-like1

The ripening process in tomato fruits is regulated by many ripening-related TFs, some of which play a positive role, such as RIN (Ito *et al.*, 2017; Li *et al.*, 2019), TDR4 (Bemer *et al.*, 2012; Fujisawa *et al.*, 2014; Zhao *et al.*, 2019), and TAGL1 (Itkin *et al.*, 2009; Vrebalov *et al.*, 2009), whereas others play a negative role, such as AP2a (Karlova *et al.*, 2011; Wang *et al.*, 2019) and SlMADS1 (Dong *et al.*, 2013). Here, we found that the ripening process of CR-NOR fruit is delayed and inhibited, while the ripening process of OE-NOR fruit is significantly accelerated (Fig. 2C). Physiological analysis of the materials revealed a significant decrease in ethylene production, carotenoid accumulation, and fruit softening in CR-NOR fruit and a significant increase in OE-NOR fruit (Fig. 3). Sequencing results showed that the expression levels of genes related to these three pathways also changed accordingly (Fig. 5). All of the data indicated that NAC-NOR is a positive regulator of tomato fruit ripening. It has been reported that four NAC family members, SlNAC1, SlNAC4, NOR-like1, and NAC-NOR, participate in tomato fruit ripening (Ma *et al.*, 2014; Zhu *et al.*, 2014; Meng *et al.*, 2016; Gao et al., 2018, 2019; Wang *et al.*, 2019). There are examples where different members of the same gene family of TFs, such as RIN and TDR4, participate in the regulation of target genes by forming oligomers (Li *et al.*, 2019). However, it is still unclear whether the four NAC gene-coding proteins interact with each other. NAC-NOR and our previously reported NOR-like1 belong to the same evolutionary clade. They have a close relationship and have 62.84% amino acid sequence identity (Gao *et al.*, 2018). However, the ripening phenotypes of the fruit in which each of these genes has been edited are significantly different. Compared with WT, CR-NOR fruit had only a 3 d delay in color break and the accumulation of pigments was slower than in WT (Fig. 2C). Ethylene production occurred, but at a reduced rate, and softening also occurred, but the mature fruit phenotype was orange-red rather than red (Gao *et al.*, 2019). In contrast, compared with the control fruit, CR-NOR-like1 fruit had a delay in color break of at least 14 d (Gao *et al.*, 2018). After color break, the production of pigments, ethylene biosynthesis, and softening were also significantly inhibited. Thus, NOR and NOR-like1 have some similar functions but also some obvious differences in the development and maturation of tomato fruits. NOR-like1 appears to be more important for fruit ripening initiation, whereas NAC-NOR has a stronger influence on carotenoid accumulation. There may not be an absolute separation of functions, however, since overexpression of *NAC-NOR* does affect fruit development and the timing of ripening.

## Supplementary data

Supplementary data are available at *JXB* online.

Dataset S1. RNA-Seq data of *nor*#11/WT, OE-NOR in WT#2/WT, and *nor*/WT.

Table S1. Primers used for qRT-PCR.

Table S2. Primers used for vector construction.

Table S3. Primers used for off-target site mutation analysis.

Table S4. Detection of mutations on putative off-target sites.

Table S5. Probes containing NACRS used in EMSA.

eraa131_suppl_Supplementary_file001

eraa131_suppl_Supplementary_file002
