# Corrigendum to: Re-evaluation of the nor mutation and the role of the NAC-NOR transcription factor in tomato fruit ripening

**DOI:** 10.1093/jxb/eraa247

**Published:** 2020-06-04

**Authors:** Ying Gao, Wei Wei, Zhongqi Fan, Xiaodan Zhao, Yiping Zhang, Yuan Jing, Benzhong Zhu, Hongliang Zhu, Wei Shan, Jianye Chen, Donald Grierson, Yunbo Luo, Tomislav Jemrić, Cai-Zhong Jiang, Da-Qi Fu

**Affiliations:** 1 Laboratory of Fruit Biology, College of Food Science & Nutritional Engineering, China Agricultural University, Beijing, China; 2 College of Agriculture & Biotechnology, Zhejiang University, Hangzhou, China; 3 State Key Laboratory for Conservation and Utilization of Subtropical Agro-bioresources/Guangdong Provincial Key Laboratory of Postharvest Science of Fruits and Vegetables, College of Horticulture, South China Agricultural University, Guangzhou, China; 4 School of Food and Chemical Engineering, Beijing Technology and Business University, Beijing, China; 5 Plant Sciences Division, School of Biosciences, University of Nottingham, Sutton Bonington Campus, Loughborough, UK; 6 Department of Pomology, Faculty of Agriculture, University of Zagreb, Zagreb, Croatia; 7 Department of Plant Sciences, University of California, Davis, CA, USA; 8 Crops Pathology and Genetics Research Unit, United States Department of Agriculture, Agricultural Research Service, Davis, CA 95616, USA

*Journal of Experimental Botany*, Advance Access publication: 27 April 2020, doi: 10.1093/jxb/eraa131

In the above article, the author list has been corrected to:

Ying Gao^1,2^, Wei Wei^3^, Zhongqi Fan^3^, Xiaodan Zhao^4^, Yiping Zhang^1^, Yuan Jing^1^, Benzhong Zhu^1^, Hongliang Zhu^1^, Wei Shan^3^, Jianye Chen^3^, Donald Grierson^2,5^, Yunbo Luo^1^, Tomislav Jemrić ^6^, Cai-Zhong Jiang^7,8^ and Da-Qi Fu^1,^*

Co-author Cai-Zhong Jiang was initially omitted from the above article. This has now been corrected. Cai-Zhong Jiang’s affiliations are as follows:

^7^Department of Plant Sciences, University of California, Davis, CA 95616, USA

^8^Crops Pathology and Genetics Research Unit, United States Department of Agriculture, Agricultural Research Service, Davis, CA 95616, USA

