# Supplementary material for: Re-evaluation of the nor mutation and the role of the NAC-NOR transcription factor in tomato fruit ripening
Source: J Exp Bot. 2020 Apr 27;71(12):3560–74. doi: 10.1093/jxb/eraa131 (PMC7307841; doi:10.1093/jxb/eraa131)
Supplement: eraa131_suppl_Supplementary_file001 [file eraa131_suppl_supplementary_file001.pdf]

Table S1. Primers used for qRT-PCR

Gene	Primer	Sequence (5'-3')
NAC-NOR	For	TCCGATTCCGGTGGATATTA
	Rev	CTCGCCCCGTTAGGATATTT
Actin	For	CAGCAGATGTGGATCTCAAA
	Rev	CTGTGGACAATGGAAGGAC
SlACS2	For	AAACCCCAACGGAGTTATCC
	Rev	GCAATGGCCTTGAATGATTT
SlACO3	For	ACCAGCTTGAGGTGATTACCAA
	Rev	GATGCTAGTGACATCCGAGTCC
SlE4	For	TCTAAATCGCCAGGGTAATGAT
	Rev	TAGCTTCTAACGACTCCCTTGC
SlERF2	For	AACGAGCTCGACCCTCTACA
	Rev	TGCAGCTTCTTCAGCAGTGT
SlGgpps2	For	GGGATTGGAAAAGGCTAAGG
	Rev	AGCAATCAATGGAGCAGCTT
SlSGR1	For	TGGCTATCTCCCAAACCATC
	Rev	GTGGCAATGGACATGAAGTG
SlPG2a	For	AGCTAAGGGTGATGGAAAAACA
	Rev	TGAAAAGGTGATTTGCTTGAGA
SlTBG4	For	TCTGCTCAGTGTTTCCGTTG
	Rev	CCATTTCTGTTTCGCCAAGT
SlCEL2	For	AAACACATTGCCAAACGTCA
	Rev	CCACAGATGGCACAGAGCTA
SlEXP1	For	AAATGACAATGGTGGCTGGT
	Rev	CTGATTCCTCCTTGCTTTCG

Table S2. Primers used for vector construction

Assay	Primer	Sequence (5’-3’)
Subcellular Localization	pEAQ-NOR-GFP-F	ctgcccaaattcgcgaccggtATGGAAAGTACGGATTCATCAACC
	pEAQ-NOR-GFP-R	tcctttgctagtcataccggtAGAGTACCAATTCATGCCAGTAACTT
	pEAQ-NOR#19-GFP	ctgcccaaattcgcgaccggtATGGAAAGTACGGATTCATCAACC
	pEAQ-NOR#19-GFP	tcctttgctagtcataccggtGCAGGGCCATGGATCAAAC
	pEAQ-NOR186-mCherry-F	ctgcccaaattcgcgaccggtATGGAAAGTACGGATTCATCAACC
	pEAQ-NOR186-mCherry-R	gcccttgctcaccataccggtTCTATGGACCTGTGTGTTATTCTTCTT
	pEAQ-RIN-mCherry-F	ctgcccaaattcgcgaccggtATGGAAAGTACGGATTCATCAACC
	pEAQ-RIN-mCherry-R	gcccttgctcaccataccggtGCAGGGCCATGGATCAAAC
Over-expression	NAC-NOR-F	acgggggactctagaggatccATGGAAAGTACGGATTCATCAACC
	NAC-NOR-R	gtacgtaaccacAGAGTACCAATTCATGCCAGTAACTT
	3×HA-NOR-F	ggtactctGTGGTTACGTACATGTATCCTTATGATG
	3×HA-NOR-R	cgatcggggaaattcgagctcCCTAGGCCCTTAAAGACTAGCATAA
EMSA	pGEX-NOR -F	ccgcgtggatccccggaattcATGGAAAGTACGGATTCATCAACC
	pGEX-NOR -R	gtcacgatgcggccgctcgagTTAAGAGTACCAATTCATGCCAGTAA
	pGEX-NOR186 -F	ccgcgtggatccccggaattcATGGAAAGTACGGATTCATCAACC
	pGEX-NOR186 -R	gtcacgatgcggccgctcgagTCATCTATGGACCTGTGTGTTATTCTT
	pGEX-NOR#19 -F	ccgcgtggatccccggaattcATGGAAAGTACGGATTCATCAACC
	pGEX-NOR#19 -R	gtcacgatgcggccgctcgagTTAGCAG

		GGCCATGGATCAA

Table S3. Primers used for off-target site mutation analysis

Gene	Off-target site	Primer	Sequence(5’-3’)
NAC-NOR	1	For	CGCACACATGGTACGAAAAA
		Rev	ACCAATGATGGCATGGATTT
		Seq	GCAGGCTAGTAGTGCGAAT
	2,4	For	CCCACCTACATCATCCATCC
		Rev	TCCTCAGCCCTGTTGGATAC
		Seq	TCCTCCCACTCTAATCTTTCTG
	3	For	GGCCAAGACGTGATCATTTT
		Rev	ACAAACCCGCATGAACTCTC
		Seq	TTCTAATGAACCCGATCCGCAAAC
	5	For	TCCAGGGTTCAGGTTTCATC
		Rev	CAGTATGACACCCCGTACGAT
		Seq	TATACCCAAAGTCAAAGGAC

Table S4. Detection of mutations on putative off-target sites

Gene	Name of putative off-target site	Putative off-target locus	Sequence of the putative off-target site	transgenic lines sequenced	No. of plants with mutations
NAC-NOR	1	SL2.50ch03:+69991761	AGTCGCAGGCCCACCGCTTCCAG	*nor*#11*3<br>*nor*#19*3	0
	2	SL2.50ch07:-65485334	CGAAACCCCGGTGGTAAATGCGG	*nor*#11*3<br>*nor*#19*3	0
	3	SL2.50ch01:-5585750	CGGCGGTTGTCGACCAAAAGTGG	*nor*#11*3<br>*nor*#19*3	0
	4	SL2.50ch07:-65485466	TTCATACCAGGAAGTTCCCAAGG	*nor*#11*3<br>*nor*#19*3	0
	5	SL2.50ch12:-51943253	TAATTACCAGGGAGGTCCCAAGG	*nor*#11*3<br>*nor*#19*3	0

Table S5. Probes containing NACRS used in EMSA

Gene	Primer	Sequence(5'-3')
SlACS2	wild-F	AATAAATTCTTTTTGACAGGGTGGCGTAAAAATAATTTTATTTTAAAATA
	wild-R	TATTTTAAAATAAAATTATTTTTACGCCACCCTGTCAAAAAGAATTTATT
	mutant-F	AATAAATTCTTTTTGACAGGGAAAAAAAAAAATAATTTTATTTTAAAATA
	mutant-R	TATTTTAAAATAAAATTATTTTTTTTTTCCCTGTCAAAAAGAATTTATT
SlGgpps2	wild-F	TACGTTTAGGATAGGACTTCATTACGTGTCAAGGACAAAAATAAACCTAT
	wild-R	ATAGGTTTATTTTTGTCCTTGACACGTAATGAAGTCCTATCCTAAACGTA
	mutant-F	TACGTTTAGGATAGGACTTCAAAAAAAAACAAGGACAAAAATAAACCTAT
	mutant-R	ATAGGTTTATTTTTGTCCTTGTTTTTTTTTGAAGTCCTATCCTAAACGTA
SlPL	wild-F	TATCAATAACAGATAGTAATTTTACGTAATGATATTGAACGATGTATATA
	wild-R	TATATACATCGTTCAATATCATTACGTAAAATTACTATCTGTTATTGATA
	mutant-F	TATCAATAACAGATAGTAATTAAAAAAAATGATATTGAACGATGTATATA
	mutant-R	TATATACATCGTTCAATATCATTTTTTTTAATTACTATCTGTTATTGATA
